# Environmental Risk Assessment of Oxaliplatin Exposure on Early Life Stages of Zebrafish (*Danio rerio*)

**DOI:** 10.3390/toxics10020081

**Published:** 2022-02-09

**Authors:** Davide Di Paola, Fabiano Capparucci, Jessica Maria Abbate, Marika Cordaro, Rosalia Crupi, Rosalba Siracusa, Ramona D’Amico, Roberta Fusco, Tiziana Genovese, Daniela Impellizzeri, Salvatore Cuzzocrea, Nunziacarla Spanò, Enrico Gugliandolo, Alessio Filippo Peritore

**Affiliations:** 1 Department of Chemical, Biological, Pharmaceutical, and Environmental Science, University of Messina, 98166 Messina, Italy; davide.dipaola@unime.it (D.D.P.); fabiano.capparucci@unime.it (F.C.); rsiracusa@unime.it (R.S.); rdamico@unime.it (R.D.); rfusco@unime.it (R.F.); tgenovese@unime.it (T.G.); dimpellizzeri@unime.it (D.I.); aperitore@unime.it (A.F.P.); 2 Department of Veterinary Science, University of Messina, 98166 Messina, Italy; jessica.abbate@unime.it (J.M.A.); rcrupi@unime.it (R.C.); egugliandolo@unime.it (E.G.); 3 Department of Biomedical and Dental Sciences and Morphofunctional Imaging, University of Messina, 98125 Messina, Italy; cordarom@unime.it; 4 Department of Pharmacological and Physiological Science, Saint Louis University School of Medicine, Saint Louis, MO 63104, USA

**Keywords:** oxaliplatin, developmental toxicity, ROS, cell death

## Abstract

Pharmaceuticals are actually identified as a threat to the ecosystem. Nowadays, the growing consumption of antineoplastic agents has been related to their continuous input in natural environments. These substances can interfere with physiological and biochemical processes of aquatic species over their entire life cycle. Oxaliplatin (OXA) is a widely used chemotherapeutic agent to treat colon or rectal cancer. This study was aimed to evaluate the developmental toxicity of the OXA exposure. To this end, zebrafish embryos were incubated with 0.001, 0.1, 0.5 mg/L OXA. At different timepoints mortality rate, hatching rate, developmental abnormalities, histological analysis, oxidative stress and mRNA expression of gene related to oxidative stress were evaluated. Our results showed that OXA exposure can induce increased mortality and developmental abnormalities reducing the hatching rate. Histological analysis demonstrated that OXA induced liver, intestine, muscle and heart injury. Superoxide dismutase and catalase activities were significantly increased after OXA exposure demonstrating its oxidative effects. The mRNA expression levels of apoptosis-related genes (caspase-3, bax and bcl-2) were significantly upregulated by OXA exposure. In conclusion, we highlighted that OXA exposure led to a dose-related developmental toxicity, oxidative stress and apoptosis.

## 1. Introduction

Global Cancer Statistics 2020 (GLOBOCAN) estimates that 19.3 million new cancer cases and about 10.0 million cancer deaths occurred globally in 2020. Data of the World Health Organization (WHO) place the use of anticancer drugs among the eight categories of medicines most employed on the planet. Platinum-based antineoplastic agents are widely used in chemotherapy. They crosslink DNA strands or form DNA-protein crosslinks in cancer cells [1,2] and include cisplatin (CDDP), carboplatin (CARP) and oxaliplatin (OXA). Several studies have shown that exposure to platinum or its derivatives, such as cisplatin, may result in genotoxic and teratogenic effects during the early stages of zebrafish embryonic development [3,4]. OXA is a chemotherapeutic agent used to treat colon or rectal cancer that has spread (metastasized) [5]. It is frequently used in conjunction with other anticancer medications (fluorouracil and leucovorin). The anticancer mode of action of platinum-complex drugs is owing to the development of platinum adjuncts between adjacent DNA bases, and it is classed as “alkylating agent”. OXA selectively inhibits the synthesis of deoxyribonucleic acid (DNA), the guanine and cytosine content correlates with the degree of OXA-induced cross-linking. At high concentrations of the drug, cellular RNA and protein synthesis are also suppressed [5]. OXA varies from cisplatin in that diaminocyclohexane replaces the amine groups in cisplatin (dach). After administration, anticancer drugs are excreted in urine and feces; OXA is mostly excreted through the kidneys: after 48 h, around half of the administered dose is detected in the urine. The liver excretes between 2 and 5% of the dosage. In patients with poor renal function, oxaliplatin clearance has been found reduced [6]. In wastewater treatment plants, human excrement is thought to be the major source of pharmaceuticals wastewater treatment plants (WWTPs) [7]. Anticancer medicines are only partially eliminated during sewage treatment methods due to their chemical features and they contaminate surface waters [8]. Recently, several studies have shown the occurrence of antitumoral drugs in the aquatic environment at ng–μg L^−1^ concentrations [9,10,11].

Numerous studies have shown increasing concentrations of platinum group elements in various parts of the water ecosystem, i.e., in drinking, ground and surface waters [12,13]. The major sources of platinum compounds in the environment are the emissions from catalytic converters of automobiles and effluents from hospitals. High levels of carcinogenic platinum compounds, such as oxaliplatin, ranging from 4.7 to 145 micrograms/liter, were observed in a study conducted on wastewater samples from hospital settings of cancer departments [11].

Pharmaceutical bioaccumulation has been documented in both laboratory [14,15,16] and field research [17,18]. Only Steinbach et al., 2013 [19] presented data on the bioaccumulation of a pharmaceutical (verapamil) in aquatic animals (*Cyprinus carpio*) during early life stages. In literature, a large amount of ecotoxicological data on anticancer drugs is present [20]. Unfortunately, there are few data on acute exposure to antitumoral drugs as environmental contaminants and their effects on the aquatic species, particularly the most vulnerable forms like larvae [21]. The Fish Embryo Toxicity (FET) test with D. rerio is a widely used protocol to assess the toxicity of environmental contaminants [22,23]. Other implications can be shown in the FET test, such as influences on developmental advancement, including as morphological deformities, delayed development, pericardial edema and yolk sac edema. In this study we evaluated the toxic effects of OXA on the development of the zebrafish embryos.

## 2. Materials and Methods

### 2.1. Solutions Preparation

OXA (Oxaliplatin SUN, 10 mL) 5 mg/mL was purchased from (SUN Pharmaceutical Industries Europe B.V. Polarisavenue 87 2132 JH Hoofddorp, Holland). The solution was diluted in embryo medium obtaining three concentrations ranging from 0.5 to 0.1 to 0.001 mg/L in 24 well plate (Labsolute, Th. Geyer GmbH & Co. KG, Dornierstr. 4–6 D-71272 Renningen, Germany), one for each concentration and one plate with negative control (untreated).

### 2.2. Zebrafish Maintenance and Breeding

Wild type (WT) mature zebrafish with an age of 6 months were used for embryos production. Zebrafish were reared in the Centre for Experimental Fish Pathology (Centro di Ittiopatologia Sperimentale della Sicilia—CISS), Department of Veterinary Sciences, University of Messina, Italy. CISS has been accredited for the use and production of aquatic models for research since 2006 and all procedures were performed according to EU/63/2010 DL. The fish were fed both with dry and live food twice a day at 3% of body weight (BW). For a successful reproduction, mature females and males were mated at 2:1 ratio. The day after, the eggs were collected, bleached and afterwards non-fertilized eggs were discarded. According to Directive 2010/63/EU and relating Italian DL 26/2014 on the protection of animals used for scientific purposes, experiments on zebrafish larvae up to five days (120 h) post fertilization and particularly ZFET are paired with alternative methods and thus they do not need ethical approval. The evaluation and authorization of projects involving the use of animals in experimental procedures refer to:

(a) Live non-human vertebrate animals, including: (i) independently feeding larval forms; and (ii) foetal forms of mammals as from the last third of their normal development;
(b) Live cephalopods.

### 2.3. Zebrafish Embryo Toxicity (ZFET) Assay

The toxicity of OXA solutions was established following the OECD guideline (OECD, Test No. 236: fish embryo acute toxicity (FET) test). Different concentrations of the OXA (0.0010–0.5 mg/L) were prepared using embryo medium and placed into 24-well plate (1 embryo each well). Fertilized eggs (*n* = 24 in each plate for each replicate of an experimental group, 20 exposed to the drug and 4 used as negative controls) were transferred into 24-well plates with test solutions and incubated at 26 °C at a 14:10 h day/night light regime. The experiment was repeated three times. The entire mortality and developmental abnormalities of embryos and larvae were monitored and recorded at 24, 48, 72 and 96 h post fertilization (hpf) [24]. Coagulation, lack of somites, non-detachment of the tail and no heartbeat was considered as lethal endpoint. Furthermore, malformations of the embryos during development was evaluated as a teratogenic endpoint. In addition, the percentage of hatching and mortality were estimated. A stereo microscope was used to capture images and movies (Leica M205 C, Leica Microsystems Srl, Buccinasco Milano, Italy). Every 24 h, four separate endpoints were checked to see any malformations:

(a) embryo coagulation—can also occur within a few hours of the start of exposure and indicates a generic acute toxic effect;
(b) lack of somite formation—somite should be visible 12 h after fertilization; if absent, the embryo will not develop further, thus causing its death;
(c) non-detachment of the tail—detachment of the tail from the yolk can be observed 24 h after fertilization, indicating normal growth of the embryo;
(d) absence of heartbeat—the heartbeat is easily detectable 30 h after fertilization, its absence indicates the death of the embryo; embryo coagulation and absence of heartbeat were focused, as endpoints of mortality.

### 2.4. Histopathological Analysis

Larvae were collected and fixed in buffered 4% paraformaldehyde for 24 h at 4 °C for histological investigation. After that, they were dehydrated, rinsed and processed in an ascending order of alcohol (70–100%), followed by xylene clearing. Paraffin wax was used to embed the samples, which were then placed on wooden blocks. Microtome was used to cut 5-μm thick, thin sections. Haematoxylin and eosin were used to stain the slides (H&E). Tissue ribbons were stretched by fixation on albumenized glass slides. Following that, the slides were inspected under a light microscope (Olympus-CX41, Olympus Italia S.r.l., Segrate, Italy).

### 2.5. Total RNA Extraction and RT-PCR

The total RNA from zebrafish larvae (10 per experimental group of each experiment) was homogenized and isolated in 0.50 mL TRIzol reagent (Invitrogen, Waltham, MA, USA) according to the manufacturer’s instructions. Total RNA was isolated according to the manufacturer’s instructions. The ratio of absorbance at 260–280 nm, as well as the banding patterns on a 1% agarose formaldehyde gel, were used to verify the quality of the RNA in each sample. RNA quality was evaluated by gel electrophoresis, with the concentration measured with NanoDrop 2000 (Thermo Scientific, Waltham, MA, USA, iScript RT-PCR kit (Bio-Rad, Hercules, CA, USA)), which was used to synthesize first-strand cDNA according to manufacturer’s recommendations. The reverse transcription master mix was prepared adding to 1 μg of RNA template the iScript RT Su-permix (5× RT supermix with RNase H+ Moloney (gray cap, 25 or 100 reactions) murine leukemia virus (MMLV) reverse transcriptase, RNase inhibitor, dNTPs, oligo (dT), random primers, buffer, MgCl_2_ and stabilizers) and the nuclease-free water. The complete reaction mix was incubated in a thermal cycler (Priming 5 min at 25 °C, Reverse transcription 20 min at 46 °C, RT inactivation for one minute at 95 °C). Real-time PCR was performed with a 20-μL volume containing 10-μL of 1× SsoFast EvaGreen Supermix (Bio-Rad, Hercules, CA, USA), 1 μL of cDNA, 7 μL of RNase/DNase-free water and 500 nM each primer. PCR conditions were initial denaturation at 95 °C for 15 min, followed by 45 cycles of amplification at 95 °C for 20 s and 60 °C for 40 s. Final extension at 60 °C for 60 s and hold at 4 °C were then performed on StepOnePlus Real-Time PCR System (Applied Biosystems, Foster City, CA, USA).The RT-PCR technique was adapted from a previous study [25]. Each gene in the present study was assessed in triplicate. The sequences of primers for the real-time PCR are shown in Table 1.

### 2.6. Cell Death and Image Analysis

The cell death percentage was obtained by assessing the fluorescence using Zou et al.’s methodology [26]. Acridine orange, a nucleic acid-specific metachromatic dye that interacts with DNA and RNA via intercalation or electrostatic attraction stains necrotic or very late apoptotic cells; thus, acridine orange staining was used to detect cell death in live zebrafish embryos. Embryos were transferred into 24-well plates at 96 hpf and treated with acridine orange for 30 min (7 μg mL^−1^), in the dark at 28 °C. The zebrafish embryos were then rinsed in fresh embryo medium five times and anesthetized before visualization. The fluorescence intensity of each zebrafish larva was quantified with the Image J program (Version 1.8.0, National Institutes of Health, Bethesda, MD, USA).

### 2.7. SOD and CAT Measurement

The larvae from each plate were defrosted and homogenized on ice with 180 μL ice-cold physiological saline. The supernatant was obtained by centrifuging the homogenate at 4000 g for 15 min at 4 °C. As previously mentioned, the concentration of SOD and CAT in the supernatant was determined using commercial kits (Nanjing Jiancheng Bioengineering Institute, Nanjing, China) [27,28,29,30,31].

### 2.8. Data Analysis

Microsoft Excel was used to evaluate all of the raw spreadsheet data. GraphPad Prism 8.3.1 software (GraphPad, San Diego, CA, USA, 2020) was used to plot and statistically analyze graphs. To find significant differences between the mean values, a two-way anova analysis of variance was utilized (ANOVA-SNK). The data were tested for normal distribution with the Kolmogorov–Smirnov test (*p* < 0.05) and they were represented as mean ± standard error of mean (SEM) (alpha value of 0.05).

## 3. Results

### 3.1. Mortality, Hatching Rate and Malformations

**Figure 1:**
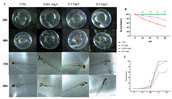
Embryo phenotypes, mortality and hatching rate after 24 to 96 h of exposure to OXA. (**A**) The embryo phenotypes in the unexposed and OXA-exposed groups. (**B**) The mortality rate in zebrafish embryos exposed to OXA. (**C**) The hatching rate in zebrafish embryos exposed to OXA. The asterisk denotes a statistically significant difference when compared with the CTRL: *** *p* < 0.001 versus control.

Figure 1A depicts the development of OXA-exposed zebrafish embryos from 24 to 96 hpf. At 96 hpf, the 0.5 mg/L OXA-treated group had many morphogenetic defects, including spinal curvature, pericardial edema and tail deformity. Figure 1B depicts the embryos cumulative survival after exposure to OXA. In order of increasing concentration, survival rate was documented at 24, 48, 72 and 96 hpf. Calculations (GraphPad Prism 8.3.1 software, San Diego, CA, USA, 2020) have been used to determine the median lethal concentration (LC50) of OXA at 96 hpf, the LC50 was 0.5 mg/L, resulting in a much higher mortality than the control group. At doses lower than 0.5 mg/L, no particular decrease in survival rate was observed (does not exceed 25% significantly); this shows that lower concentrations do not have a major impact on zebrafish larvae development. Because hatching is a critical time in zebrafish embryogenesis, the hatching rate is one of the most important indices for determining OXA developmental toxicity in zebrafish. According to the studies conducted, embryos started to hatch by 48 hpf and finished by 72 hpf. Our results showed that 76% of the 0.001 mg/L group all embryos had hatched by 72 hpf, while in groups with concentrations of 0.1 mg/l and 0.5 mg/l, the hatching rate was 75% and 61% respectively. Therefore, as shown in Figure 1C, the embryo-hatching rate was reduced in the OXA higher concentrations (0.1–0.5 mg/L), mostly in the highest dose treated group (0.5 mg/L). These data showed a slight dose-dependent decrease in the hatching rate in the 0.1 and 0.5 mg/L OXA-treated groups compared with the control group (100% hatching at 72 hpf).

### 3.2. OXA Effect on Stress Oxidative Pathway

**Figure 2:**
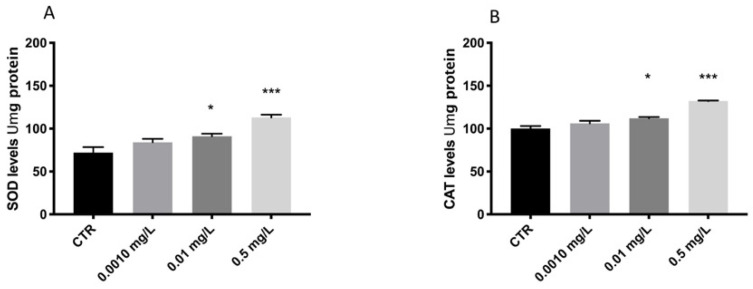
Effects of OXA exposure on activities of SOD (**A**) and CAT (**B**), in the larval zebrafish. Embryonic zebrafish was exposed to OXA for 96 hpf. Data are expressed as the mean ± SEM of three replicates (about 10 larvae per replicate). The asterisk denotes a statistically significant difference when compared with the CTRL: * *p* < 0.05, *** *p* < 0.001 versus control.

The results showed an increase of SOD and CAT expression levels related to oxidative damage after OXA exposure at high doses (0.1 and 0.5 mg/L) (Figure 2). Contrarily, OXA at 0.001 mg/L did not show effect on stress oxidative in larval compared to CTRL group (Figure 2).

### 3.3. Histological Analysis

**Figure 3:**
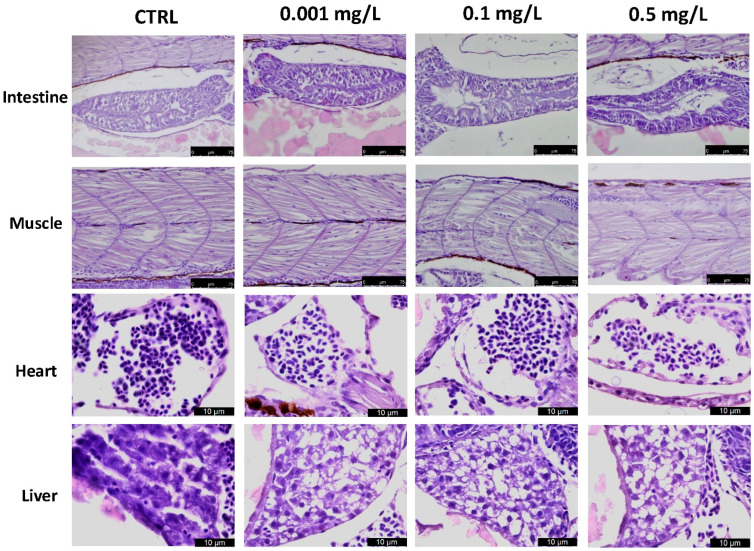
Histopathological changes in the hearts, livers, intestines, and muscles of zebrafish larvae exposed to OXA at 96 hpf. LV = Liver; HE = Heart. Data are presented as means ± SEM or medians with interquartile ranges for non-parametric data of 10 larvae for each group. Scale bars 40× magnification.

OXA produced noticeable effects in a dose dependent manner on the heart, liver, intestines and muscle, including mild fat liver degeneration with atrophy at 96 hpf, frayed gut villi with epithelial desquamation, as depicted in Figure 3. The OXA group at 0.001 mg/L showed no significant histological alteration compared to the CTRL group, while the 0.1 groups and even more the group with the higher dose (0.5 mg/L of OXA) showed histological changes (more than 50% of the entire group) in intestine, infiltrate and edema in heart and muscle and also hepatocytes loss in liver.

### 3.4. Cell Death Process

**Figure 4:**
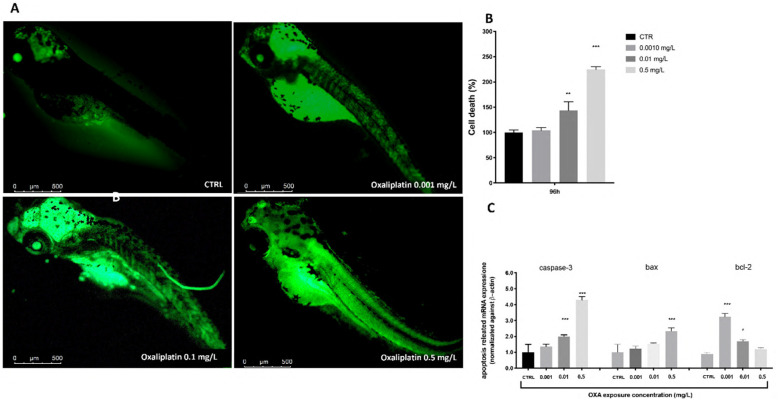
The OXA exposure effects on cell death zebrafish embryos. At 96 hpf, OXA 0.001, 0.1, 0.5 mg/L exposure, the levels of cell death were observed and photographed by a fluorescence microscope after staining with acridine orange (**A**). Percentage of cell death histogram (**B**). The results are expressed as mean of three independent experiment data. Related gene expression levels of apoptotic pathway in zebrafish embryos exposed to OXA at concentrations of 0.001, 0.1, 0.5 mg/L at 48 hpf (**C**). The fold change from the CTRL group is used to reflect the mRNA expression levels. * *p* < 0.05, ** *p* < 0.01, *** *p* < 0.001 versus control. Scale bars 4× magnification.

Enhanced fluorescence intensities were clearly observed in OXA-exposed zebrafish larvae when compared to the CTRL. The cell death was forcefully increased in a dose-dependent manner after OXA exposure. This value was reduced remarkably at 0.001 mg/L compared to the 0.1 and 0.5 mg/L group. The results indicate that OXA in a dose dependent manner induced cell death in zebrafish embryos. Moreover, we employed RT-PCR to assess the mRNA expression levels of larvae exposed to different OXA concentrations at 96 hpf to determine the possible causes of the harmful effects generated by OXA. The expression levels of apoptosis-related genes (caspase-3 and bax) rose with increasing OXA exposure dose, according to the RT-PCR data. With increasing OXA exposure levels, bcl-2 mRNA expression was downregulated(Figure 4).

## 4. Discussion

Several studies have already highlighted the presence of different typologies of drugs in aquatic environments [32]. The development of environmental quality standards and/or guidelines for pharmaceuticals in seawater, sediments and biota would be aided by the expansion of data on the ecological consequences of pharmaceuticals present in the examined environment. The proposed short-term approaches to minimize the exposure of humans, animals and ecosystems to these substances include extensive monitoring of pharmaceuticals in the environment and their influence on living species [33,34,35]. In this manuscript we investigated the effects of OXA exposure at three different concentrations. At 0.001 mg/L, OXA treatments did not show any noticeable signs of toxicity, whereas doses of 0.1 and 0.5 mg/L significantly affected hatching rate and zebrafish embryo development. It has been widely demonstrated that OXA therapy caused embryonic teratogenesis, which included pericardial edema and spinal malformation. The most prevalent type of malformation, spinal cord teratogenesis, may be linked to an ion imbalance (such as calcium and phosphorus) or a decrease in myosin, which are both required for development [36,37]. As already demonstrated in other studies, OXA is able to interfere and block protein synthesis in a dose-dependent manner [5]. For this reason, it is very plausible to consider this mechanism of action also involved in the defections found in the development of the larvae and at the same time in their hatching rate. Because hatching is such an important stage in zebrafish embryogenesis, the lower hatching rate was attributed to structural and functional disruptions that took place during the embryonic stage [38,39]. In addition, the difficulty of emerging larvae to break the eggshell [40] or the inhibition of mitosis or embryogenesis [41] also played a role in the developmental delay. Our research highlighted a slight dose-dependent drop-in hatching rate. Moreover, this delay in hatching is also a good indicator of developmental toxicity. According to the study performed, OXA exposition at moderate doses caused embryonic teratogenesis, which included pericardial edema, spinal curvature, uninflated swim bladders and twisted tails. Histological analysis of zebrafish larvae confirmed the detrimental effects of OXA exposure showing heart, liver, intestine and muscle injury. Several studies reported that drug-induced oxidative stress has two basic mechanisms: an increase in ROS generation and a loss in cellular antioxidant defenses [42,43]. The main cause of oxidative stress is reactive oxygen species (ROS). Excessive production of ROS in vivo causes oxidative stress [44], since oxygen free radicals can react excessively with SOD and CAT, causing an imbalance in the body’s antioxidant protection mechanism. SOD is an antioxidant enzyme that can protect the body from oxidative damage caused by the environment, eliminate ROS and prevent lipid peroxidation [31]. The ability of antioxidant defenses was diminished in the high-exposure group, which could be because negative feedback regulation is important in low concentration stimulation. These findings showed that oxidative stress was triggered in OXA-exposed zebrafish embryos and had a key role in OXA developmental toxicity. Several studies have largely highlighted the involvement of oxidative stress in the activation of apoptosis processes [45,46]. These correlations between oxidative damage and cell death have also been demonstrated in several studies conducted in both larvae and adult zebrafish [47,48]. Moreover, involvement of OXA in triggering of apoptosis mechanisms has already been documented in literature [49,50,51]. In the present study, we demonstrated how exposure to a dose of 0.5 mg/L of OXA not only causes an imbalance in antioxidant defenses but also results in an increase in markers related to the apoptotic process. In fact, exposure to OXA increased both the levels of apoptosis-related genes (caspase-3 and bax) and a downregulation of the mRNA expression of anti-apoptotic gene bcl-2. This increase in apoptotic markers is also accompanied by an increase in the percentage of cell death seen in OXA-exposed larvae.

## 5. Conclusions

Finally, OXA induced a dose-dependent increase in developmental harm in zebrafish embryos, as demonstrated by an increase in mortality and deformities and a delay in hatching. The information gathered in this study will aid in deciphering the mechanisms of OXA-induced developmental harm.

## Figures and Tables

**Table 1:** Primers for real-time PCR.

Gene	Primer Orientation	Nucleotide Sequence
*b-actin*	forward	5′-AGAGCTATGAGCTGCCTGACG-3′
	reverse	5′-CCGCAAGATTCCATACCCA-3′
*casp-3*	forward	5′-CCGCTGCCCATCACTA-3′
	reverse	5′-ATCCTTTCACGACCATCT-3′
*Bax*	forward	5′-GGCTATTTCAACCAGGGTTCC-3′
	reverse	5′-TGCGAATCACCAATGCTGT-3′
*bcl-2*	forward	5′-TCACTCGTTCAGACCCTCAT-3′
	reverse	5′-ACGCTTTCCACGCACAT-3′

## Data Availability

Not applicable.
