# Correction: Di Paola et al. Environmental Risk Assessment of Oxaliplatin Exposure on Early Life Stages of Zebrafish (*Danio rerio*). *Toxics* 2022, *10*, 81

**DOI:** 10.3390/toxics13010028

**Published:** 2024-12-31

**Authors:** Davide Di Paola, Fabiano Capparucci, Jessica Maria Abbate, Marika Cordaro, Rosalia Crupi, Rosalba Siracusa, Ramona D’Amico, Roberta Fusco, Tiziana Genovese, Daniela Impellizzeri, Salvatore Cuzzocrea, Nunziacarla Spanò, Enrico Gugliandolo, Alessio Filippo Peritore

**Affiliations:** 1 Department of Chemical, Biological, Pharmaceutical, and Environmental Science, University of Messina, 98166 Messina, Italy; davide.dipaola@unime.it (D.D.P.); fabiano.capparucci@unime.it (F.C.); rsiracusa@unime.it (R.S.); rdamico@unime.it (R.D.); rfusco@unime.it (R.F.); tgenovese@unime.it (T.G.); dimpellizzeri@unime.it (D.I.); aperitore@unime.it (A.F.P.); 2 Department of Veterinary Science, University of Messina, 98166 Messina, Italy; jessica.abbate@unime.it (J.M.A.); rcrupi@unime.it (R.C.); egugliandolo@unime.it (E.G.); 3 Department of Biomedical and Dental Sciences and Morphofunctional Imaging, University of Messina, 98125 Messina, Italy; cordarom@unime.it; 4 Department of Pharmacological and Physiological Science, Saint Louis University School of Medicine, Saint Louis, MO 63104, USA

## Error in Figure

In the original published publication [1], there was a mistake in Figure 3. Given the animal’s small size and anatomy, the magnified images of the heart and liver appeared in multiple boxes. The authors have indicated only the organ in question for each box, providing a clearer view of the selected organ. The corrected Figure 3 appears below. The authors state that the scientific conclusions are unaffected. This correction was approved by the Academic Editor. The original publication has also been updated.

## Figures and Tables

**Figure 3:**
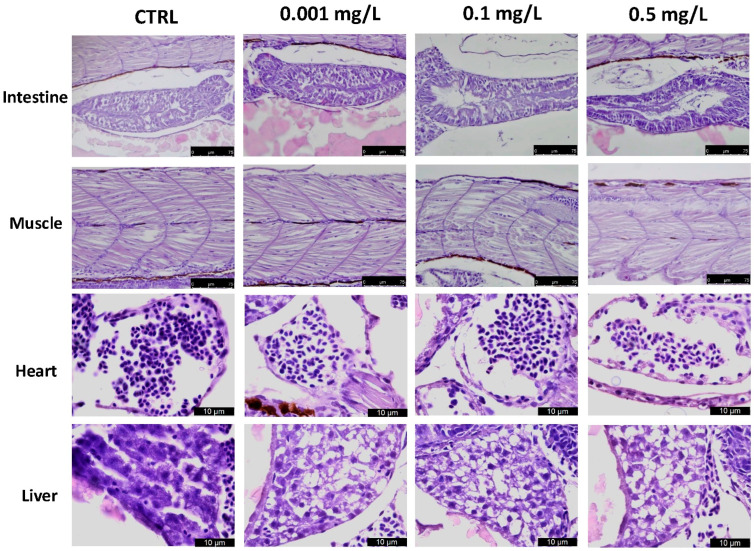
Histopathological changes in the hearts, livers, intestines, and muscles of zebrafish larvae exposed to OXA at 96 hpf. LV = Liver; HE = Heart. Data are presented as means ± SEM or medians with interquartile ranges for non-parametric data of 10 larvae for each group. Scale bars 40× magnification.
